# Catheter-Obtained Urine Culture Contamination Among Young Infants: A Prospective Cohort Study

**DOI:** 10.3389/fped.2021.762577

**Published:** 2021-11-01

**Authors:** Hilla Bahat, Revital Apelman Cipele, Tali Maymon, Ilan Youngster, Michael Goldman

**Affiliations:** ^1^Department of Pediatrics, Shamir Medical Center, Zerifin, Israel; ^2^Sackler School of Medicine, Tel Aviv University, Tel Aviv, Israel

**Keywords:** urine culture, contamination, infant, catheter, urinary tract infection

## Abstract

**Objectives:** A correct diagnosis of urinary tract infection in young infants requires an uncontaminated urine culture, commonly obtained by urethral catheterization. In the current study, we examined the rates and factors associated with contaminations of catheter-obtained urine cultures in very young infants.

**Methods:** This prospective cohort study included 143 catheter-obtained urine cultures of infants ≤2 months of age admitted to the pediatric ward of a tertiary hospital in Israel from April 2019 to September 2020. Patient's and operator's study variables were documented at the time of catheter insertion. Positive urine cultures were reviewed by a pediatric nephrologist and a pediatric infectious disease specialist and designated as infection or contamination. The study variables were compared between those with or without contamination.

**Results:** The contamination rate in our cohort was 29%. Females were more than twice as likely to have a contaminated urine culture (37 vs. 18%, respectively, *P* = 0.014). Circumcision status, official training about sterile catheterization, a sense of difficult catheterization, and the shift in which the culture was obtained did not influence the contamination rate.

**Conclusions:** Catheter-obtained urine cultures have a high contamination rate among very young infants, especially among girls.

## Introduction

Urinary tract infection (UTI) is the most common bacterial infection in febrile infants younger than 2 months of age (1), and thus, urine should be evaluated as a part of their workup. A correct diagnosis of UTI requires an uncontaminated urine sample for culture, while improper urine collection can cause under-diagnosis without proper treatment and workup or over-diagnosis resulting in unnecessary interventions.

Suprapubic aspiration (SPA) has been considered the gold-standard technique for obtaining a sterile urine culture without contamination by the perineal flora (2), but it is considered unacceptably invasive and painful (3), requires technical expertise and experience, and has a variable success rate (2, 4, 5). Thus, in our hospital, as well as in many pediatric facilities, in incontinent children, a urine culture is obtained by transurethral catheterization—a less painful method with a high specificity and sensitivity for diagnosing UTI (2). According to the 2011 American Academy of Pediatrics (AAP) guidelines for the Diagnosis of UTI (among older infants), UTI diagnosis requires an abnormal urinalysis and the presence of ≥50,000 colony-forming units (CFUs) per ml of a single urinary pathogen cultured from a urine specimen obtained through SPA or catheterization, and the culture is considered contaminated if it grows two or more urinary pathogens or a pathogen unlikely to cause UTI (2). Definitions of positive or negative culture results among very young infants are not absolute. Urine dipstick has a low sensitivity for UTI diagnosis among febrile 0–2 month-old infants (6–8), and a lower threshold of ≥10,000 CFUs per ml of a single urinary pathogen from a catheter-obtained urine culture is used by some researchers to diagnose UTI (6, 8). Catheter-obtained urine culture can be technically challenging in small infants, girls, and uncircumcised boys, and the reported contamination rate by this technique is 1.6–29% (4, 9–13).

In the current study, we prospectively evaluated the contamination rate of catheter-obtained urine cultures of infants ≤2 months of age admitted to the pediatric ward and the effect of patient-associated and operator-associated variables on the contamination rate.

## Materials and Methods

In this prospective cohort study, we collected information about catheter-obtained urine cultures from infants ≤2 months of age admitted to a single pediatric ward of a large tertiary hospital, from April 2019 to September 2020.

The pediatric ward physicians and nurses were requested to document all catheter-obtained specimens of infants ≤2 months of age sequentially.

### Urine Sample Collection

The urine culture is obtained by two team members—either a nurse and a physician or two nurses. After cleaning of the perineum with an antiseptic solution, a 5F sterile feeding tube tip is immersed in sterile lubricating jelly and inserted into the urethra. The urine is collected in a sterile container and sent to the lab immediately for urinalysis and culture.

Preferably, the first few urine drops are discarded, unless there was a very small amount of urine for culture.

At the time of the procedure, the attending nurse documented the following parameters:

Patient: Sex, age, indication for urine culture, circumcision, abnormal genitals, and urine dipstick (abnormal if positive leukocytes and/or nitrite).

Operator: Attendance in official training about sterile technique for catheter-obtained urine culture, difficult catheter insertion that was defined subjectively by the attending nurse, and shift (morning/evening/night).

After the patient's discharge, a pediatric nephrologist reviewed the final diagnosis from the discharge summary and the urine culture results and, if in doubt, consulted a pediatric infectious disease specialist. A positive culture was defined as growth of a single urinary pathogen ≥10,000 CFU/ml (6, 8). Due to the low sensitivity of urine dipstick in this age group (6–8), the urine culture was flagged as negative, positive, or contaminated irrespective of the urine dipstick results.

Contamination was defined as the growth of two or more urinary pathogens or a pathogen unlikely to cause UTI in an immunocompetent host.

Cultures growing <10,000 CFU/ml of a single urinary pathogen were excluded, due to a lack of a consensus about their significance (14).

Sterile urine cultures were defined as negative.

The inclusion criteria include catheter-obtained urine cultures from infants ≤2 months admitted to a single pediatric ward.

The exclusion criteria include genital malformation, urine culture obtained outside the pediatric ward, urine culture not obtained by catheter, urine culture taken without documentation of the abovementioned parameters, and urine cultures growing <10,000 CFU/ml of a single urinary pathogen.

### Statistical Analysis

Age was evaluated for normal distribution using the Kolmogorov–Smirnov test. Since age was not normally distributed, it was reported as median and interquartile range. Categorical variables were compared between those with or without contamination using chi-square test or Fisher's exact test. Age was compared between those groups using the Mann–Whitney test. All statistical tests were two-sided, and *P* < 0.05 was considered significant. SPSS software was used for all statistical analysis (IBM SPSS Statistics for Windows version 24, IBM Corporation, Armonk, NY, USA, 2016).

The study was approved by the institutional review board and deemed exempt from informed consent.

## Results

**Figure 1:**
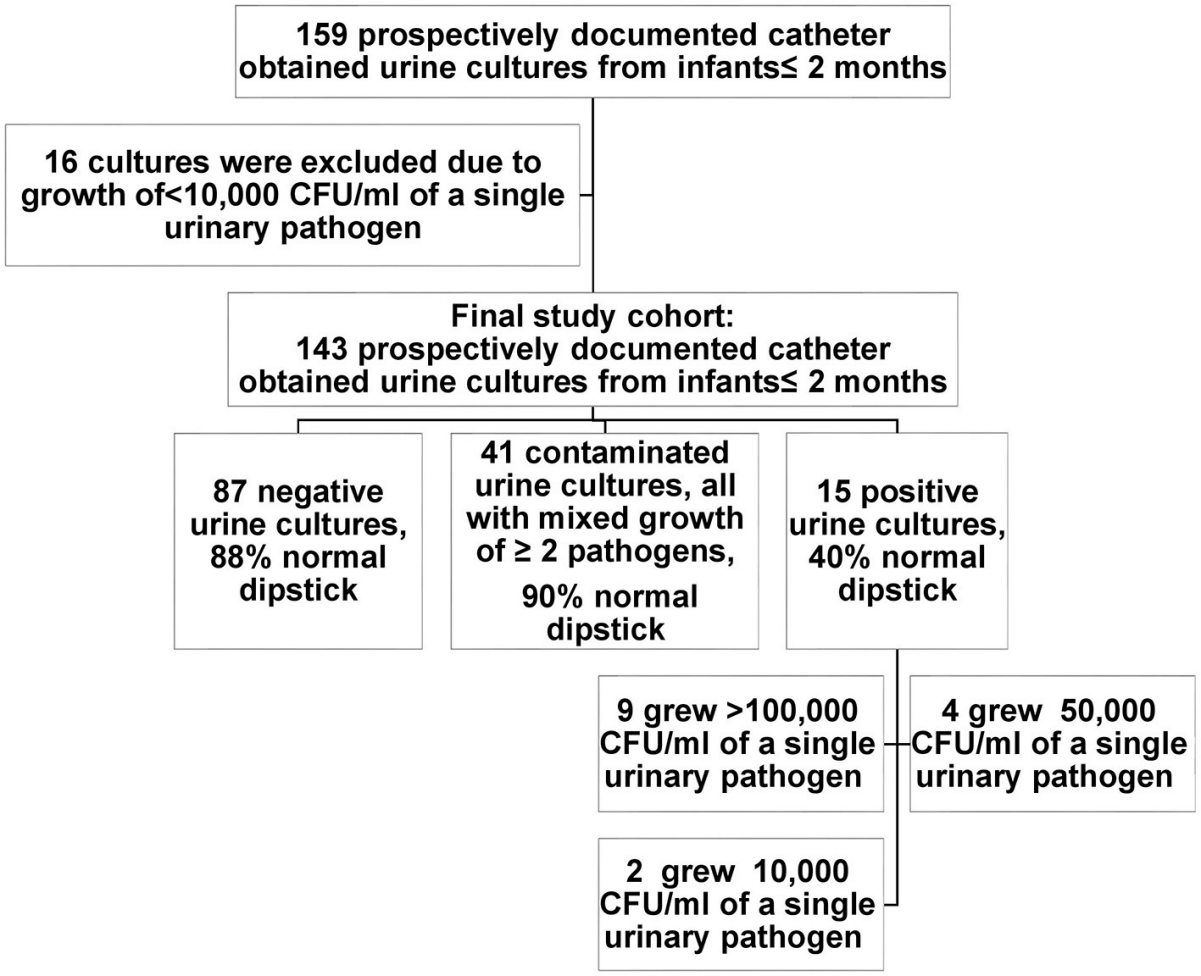
Dipstick and urine culture characteristics of the study cohort.

The final cohort included 143 infants who had catheter-obtained urine cultures, after excluding 16 cultures due to bacterial growth of <10,000 CFU/ml (Figure 1). All infants had a single urine culture. The cohort included 65 males, of which 52 were circumcised (80%). The median age was 4 weeks (IQR 3–5.5). The most common indication for urine culture was fever (67%). The cohort characteristics are summarized in Table 1.

**Table 1:** Characteristics of the study cohort.

	**Number (%)**
**Patient's gender**	
Male	65 (45%)
Female	78 (55%)
**Fever**	96 (67%)
**UTI**	17 (12%)
**Difficult catheterization**	30 (21%)
**Circumcised males**	52 (80%)
**Shift**	
Morning	43 (30%)
Evening	46 (32%)
Night	54 (38%)
**Contamination**	41 (29%)

*UTI, urinary tract infection*.

According to the discharge summary, UTI was diagnosed in 17 infants (12%), 11 (65%) of them having an abnormal urine dipstick. Two out of the 17 that were summarized and treated as UTI in the pediatric ward had a contaminated urine culture. Fifteen urine cultures were flagged as positive (10%), 87 were flagged as negative (61%), and 41 were flagged as contaminated (29%). In the positive urine culture group, nine cultures grew >100,000 CFU/ml of a single urinary pathogen [*Escherichia coli* (7), *Klebsiella pneumoniae* (1), and *Citrobacter freundii* (1)], four cultures grew 50,000 CFU/ml of a single urinary pathogen [*E. coli* (2) and *Staphylococcus aureus* (2)], and two grew 10,000 CFU/ml of a single urinary pathogen [*E. coli* (1) and *Enterococcus faecalis* (1)]. All the contaminated cultures showed a mixed growth of ≥2 pathogens. None had a growth of pathogen unlikely to cause UTI. Urine dipstick was normal in 37 (90%).

Eighty-six percent of the operators had undergone official training about sterile technique for catheter-obtained urine culture. Thirty percent of the cultures were obtained in the morning shift, 32% in the evening, and 38% at night.

Urine culture contamination was significantly more common in female infants compared to males (37 vs. 18%, respectively, *P* = 0.014). Circumcision status, official training about sterile technique for catheter-obtained urine culture, a sense of difficult catheterization, and the shift in which the culture was taken were not associated with the contamination rate (Table 2).

**Table 2:** Comparison between the groups with or without contamination.

	**No contamination**	**Contamination**	***P*-value**
**Gender**			
Male	53 (82%)	12 (18%)	*P* = 0.014
Female	49 (63%)	29 (37%)	
**Training**			
Yes	85 (71%)	35 (29%)	*P* = 0.8
No	14 (74%)	5 (26%)	
**Difficult catheterization**			
Yes	18 (60%)	12 (40%)	*P* = 0.12
No	84 (74%)	29 (26%)	
**Circumcision**			
No	9 (69%)	4 (31%)	*P* = 0.24
Yes	44 (85%)	8 (15%)	
**Shift**			
Morning	31 (72%)	12 (28%)	*P* = 0.33
Evening	36 (78%)	10 (22%)	
Night	35 (65%)	19 (35%)	

## Discussion

In this prospective study, we found a high contamination rate of catheter-obtained urine cultures, of 29% among sick infants ≤2 months of age admitted to the pediatric ward.

In most previous studies, the reported contamination rate was lower, but the study design, definition of a positive urine culture, interpretation of low bacterial count, rate of circumcision, indication for the test, and the patient's age group were variable (4, 9–11, 13, 15). In a retrospective study among older infants (2–24 months of age) in our pediatric department, we found a lower catheter-obtained urine culture contamination rate of 17%: 14% among boys and 18% among girls (unpublished data), emphasizing the higher contamination risk in very young infants.

Rivas-García et al. showed an extremely low contamination rate of catheter-obtained urine cultures (1.6%) and clean catch urine cultures (6.8%) in a retrospective study among infants <3 months visiting a pediatric emergency room. The cultures were obtained by nurses after a training course, and cultures with low bacterial counts were considered as negative, a fact that can decrease the calculated contamination rate (13). No other study has showed such a low contamination rate. In two small prospective studies among neonates, the contamination rate of catheter-obtained urine culture was 10–22% (4, 15). In agreement with our results, Wingerter et al. showed the same contamination rate among infants <6 months, while using the same definition of contamination (12).

We found that urine culture contamination was significantly more common in girls and that the contamination rate among boys was not influenced by circumcision, while Wingerter et al. found that the risk of contamination was highest among uncircumcised boys (12). This might be explained by the high frequency of ritual circumcision in our population, usually performed on the eighth day of life, and the small number of uncircumcised boys in our cohort.

Urine culture contamination is associated with adverse clinical outcomes, including delayed diagnosis and treatment of pyelonephritis on one hand and unnecessary treatment, radiologic evaluation, and hospital admission on the other hand (10). All urine culture collection methods for non-toilet-trained children have drawbacks. Collection bags and clean catch are non-invasive methods, but with extremely high contamination rates (2, 9–11, 15) and eventually a higher cost than other urine collection methods, taking into consideration equipment, staff time, and time occupying a hospital bed (5).

SPA, the gold-standard technique for obtaining a sterile urine culture with the lowest contamination rate, is considered unacceptably invasive and painful (3), with a relatively low success rate (4, 5). Catheterization is less invasive and painful compared to SPA (3), its success rate is higher (4, 5), and it has a lower contamination rate than collection bags and clean catch urine culture (5, 9–11, 15). Furthermore, catheterization is the most cost-effective urine collection method among non-toilet-trained children when compared to urine bag, clean catch, and SPA (5). Thus, catheterization is the preferred urine culture method in our hospital and in many other pediatric departments.

The high contamination rate among very young infant should be addressed, and efforts should be made to decrease it. If possible, the first few urine drops should be discarded prior to collecting the sample in the sterile container, especially in girls. This can be challenging when a very small amount of urine is obtained for urinalysis and culture. Furthermore, due to the technical challenge in this young age group, catheterizations should preferably be performed by an experienced personnel.

Our study has some limitations. To the best of our knowledge, this is the largest prospective study, assessing patient-associated and operator-associated factors influencing urine contamination rate in very young infants. However, our sample size might be too small to detect the effect of circumcision, difficulty of catheterization, training, and the timing of the procedure on the contamination rate. Although the pediatric ward staff was requested to document sequentially all the catheter-obtained urine cultures, it is possible that some specimens were not documented and missed.

This is a single-center study based on our local practices. Although our contamination rate was higher than reported in most previous studies, we suspect our data reflects the unique age group of very young infants, and not a feature specific to our center, since the contamination rate in older infants in our department is significantly lower. Furthermore, the small number of documented uncircumcised males makes our results less valid in populations with a low circumcision rate. Finally, there might be a potential bias due to the various levels of experience among the personnel inserting the catheter.

## Conclusion

Catheter-obtained urine cultures have a high contamination rate among admitted infants ≤2 months of age. The contamination risk was significantly higher among girls.

## Data Availability Statement

The raw data supporting the conclusions of this article will be made available by the authors, without undue reservation.

## Ethics Statement

The studies involving human participants were reviewed and approved by Institutional Review Board, Shamir Medical Center. Written informed consent from the participants' legal guardian/next of kin was not required to participate in this study in accordance with the national legislation and the institutional requirements.

## Author Contributions

HB designed the study, participated in data acquisition and analysis, drafted the manuscript, and approved the final version to be submitted. RAC and TM participated in data acquisition, revised the manuscript critically, and approved the final version to be submitted. IY and MG participated in the study concept, revised the manuscript critically, and approved the final version to be submitted.

## Conflict of Interest

The authors declare that the research was conducted in the absence of any commercial or financial relationships that could be construed as a potential conflict of interest.

## Publisher's Note

All claims expressed in this article are solely those of the authors and do not necessarily represent those of their affiliated organizations, or those of the publisher, the editors and the reviewers. Any product that may be evaluated in this article, or claim that may be made by its manufacturer, is not guaranteed or endorsed by the publisher.
